# CHA_2_DS_2_ ‐VASc Score as a Predictor for Atrial Fibrillation Recurrence and Clinical Outcomes Following Pulmonary Vein Isolation

**DOI:** 10.1111/anec.70088

**Published:** 2025-05-08

**Authors:** Mustafa Gabarin, Mahmoud Suleiman, Adi Elias, Ibrahim Marai, Roy Beinart, Eyal Nof, Yoav Michowitz, Michael Glikson, Yuval Konstantino, Moti Haim, David Luria, David Pereg, Avishag Laish‐Farkash, Alexander Omelchenko, the Israeli Working Group on Pacing Electrophysiology

**Affiliations:** ^1^ Cardiology Department, Meir Medical Center, Kfar‐Saba and Sackler Faculty of Medicine Tel‐Aviv University Tel Aviv Israel; ^2^ Eyal Ofer Heart Hospital, Cardiac Electrophysiology and Pacing Rambam Health Care Campus Haifa Israel; ^3^ Rappaport Faculty of Medicine Technion‐Israel Institute of Technology Haifa Israel; ^4^ Cardiology Department, Baruch Padeh Medical Center, the Azrieli Faculty of Medicine in the Galilee Bar‐Ilan University Safed Israel; ^5^ Heart Institute, Sheba Medical Center, Tel‐Hashomer, Israel and Sackler School of Medicine Tel Aviv Israel; ^6^ Jesselson Integrated Heart Center Shaare Zedek Medical Center and Hebrew University Jerusalem Israel; ^7^ Department of Cardiology, Cardiac Electrophysiology and Pacing Soroka University Hospital, ben‐Gurion University of the Negev Beer‐Sheva Israel; ^8^ Department of Cardiology, Hadassah Medical Organization and Faculty of Medicine Hebrew University of Jerusalem Jerusalem Israel; ^9^ Department of Cardiology, Electrophysiology and Pacing Unit Assuta Ashdod University Medical Center, ben‐Gurion University of the Negev Ashdod Israel

**Keywords:** AF recurrence, atrial fibrillation (AF), CHA_2_DS_2_‐VASc score, pulmonary vein isolation (PVI)

## Abstract

**Background:**

Atrial fibrillation (AF) is the most common sustained cardiac arrhythmia in adults, associated with serious cardiovascular complications such as ischemic stroke, heart failure, and myocardial infarction. Pulmonary vein isolation (PVI) is an established rhythm‐control strategy for AF. Although the CHA_2_DS_2_ ‐VASc score is primarily used to estimate stroke risk in patients with AF, its potential utility in predicting AF recurrence after PVI has not been fully explored in contemporary, real‐world multicenter settings.

**Aim:**

To evaluate the association between the CHA_2_DS_2_ ‐VASc score and both AF recurrence and adverse clinical outcomes following PVI.

**Methods:**

We conducted a retrospective cohort study using the Israeli Catheter Ablation Registry (ICAR), including 860 patients undergoing their first PVI for AF. Patients were grouped by CHA_2_DS_2_ ‐VASc score (0–1, 2–4, > 5). The primary endpoint was AF recurrence within 12 months. Secondary endpoints included re‐hospitalization, major adverse cardiovascular events (MACE), and all‐cause mortality.

**Results:**

AF recurrence occurred in 32% of patients. Recurrence rates were 25.7%, 31.4%, and 51% across the low, intermediate, and high CHA_2_DS_2_ ‐VASc score groups, respectively. A higher score was independently associated with increased recurrence risk (HR = 2.88; 95% CI, 1.75–4.74; *p* < 0.001). Elevated CHA_2_DS_2_ ‐VASc scores also correlated with higher MACE and re‐hospitalization rates. No significant difference in all‐cause mortality was observed.

**Conclusion:**

The CHA_2_DS_2_ ‐VASc score is an independent predictor of AF recurrence and adverse outcomes after PVI. Its simplicity, availability, and routine use make it a clinically useful tool to support preprocedural risk stratification in AF patients undergoing ablation.

## Graphical abstract

The CHA_2_DS_2_‐VASc score independently predicts atrial fibrillation recurrence and adverse outcomes after pulmonary vein isolation. Its routine clinical use offers a simple tool to support risk stratification and personalized post‐ablation care.

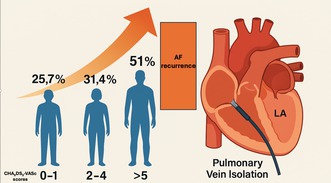

## 1 Introduction

Atrial fibrillation (AF) is the most prevalent sustained cardiac arrhythmia in adults and is associated with an increased risk of ischemic stroke, heart failure, and mortality (Benjamin et al. 2019; Hindricks et al. 2021; Wang et al. 2003). Pulmonary vein isolation (PVI) is a well‐validated treatment option for rhythm control in patients with AF, especially when performed in experienced centers (Krittayaphong et al. 2003; Stabile et al. 2006; Packer et al. 2013). However, procedural success varies across populations and depends on clinical and structural predictors, including AF type, left atrial dimensions, and comorbidities (Tzeis et al. 2024).

The CHA_2_DS_2_‐VASc score, originally developed to estimate stroke risk in non‐valvular AF, has also been linked to other adverse cardiovascular outcomes, including myocardial infarction and heart failure (Ruff et al. 2014; Proietti et al. 2020; Gabarin et al. 2022; Ruddox et al. 2017). Several studies have examined its association with AF recurrence after ablation, but most have been limited by small sample sizes, older ablation techniques, or single‐center designs (Letsas et al. 2014; Kornej et al. 2014).

Given the growing use of PVI in diverse clinical settings, there is a need for simple, widely accessible tools to help guide patient selection and risk stratification. While advanced predictors such as left atrial volume or fibrosis imaging may offer precision, they are often unavailable in routine practice. In contrast, the CHA_2_DS_2_‐VASc score offers an immediate, bedside estimate of baseline risk.

In this study, we investigated the association between the CHA_2_DS_2_‐VASc score and procedural success, defined by AF recurrence and adverse clinical outcomes, using real‐world data from a contemporary, multicenter national registry of patients undergoing their first PVI. This analysis aims to clarify whether this commonly used clinical score can serve as a practical predictor of outcomes beyond its original role in stroke risk estimation.

## 2 Methods

This retrospective cohort study is based on The Israeli Catheter Ablation Registry (ICAR), a national multicenter prospective observational registry that included patients who underwent any type of AF catheter ablation between 2021 and 2022. Study physicians recorded all clinical and demographic data, including antiarrhythmic drug (AAD) treatments and PVI procedural details. The diagnosis and classification of AF were based on clinical and electrocardiographic criteria, with patient management left to the discretion of each medical center. The study follow‐up period was 12 months, during which heart rhythm monitoring was conducted according to each center's practice. Data regarding AF recurrence and other clinical events were collected from medical records and through telephone interviews.

The CHA_2_DS_2_‐VASc score was calculated for each patient, and the study population was divided into three groups accordingly: scores of 0–1, 2–4, and > 5. The primary clinical outcome of this study was the first recurrence of AF based on the CHA_2_DS_2_‐VASc score. Secondary outcomes included re‐hospitalization, all‐cause mortality, and major adverse cardiac events (MACE), which consisted of a composite of cardiovascular death, acute myocardial infarction, and stroke/transient ischemic attack. The study adhered to the principles outlined in the Declaration of Helsinki, with all patients providing informed consent prior to enrollment. The study protocol was approved by the ethics committee at each participating center.

### 2.1 Statistical Analysis

Descriptive statistics outlining the characteristics of the study population were provided, presenting frequencies and percentages for categorical variables, as well as mean (standard deviation) or median (interquartile range) for continuous variables with normal or non‐normal distributions, respectively. Additionally, the tables included information on the percentage of missing data for transparency and completeness. To compare categorical variables across the groups, a chi‐square test for trend was utilized. For normally distributed continuous variables, an analysis of variance (ANOVA) with one degree of freedom was performed, while Kendall rank correlation was used for non‐normally distributed continuous variables. The Kaplan–Meier method was employed to analyze the time to the first occurrence of an AF event within a one‐year period. In the multivariate analysis, variables with a *p* < 0.05 in the univariate models were selected for testing.

## 3 Results

**FIGURE 1:**
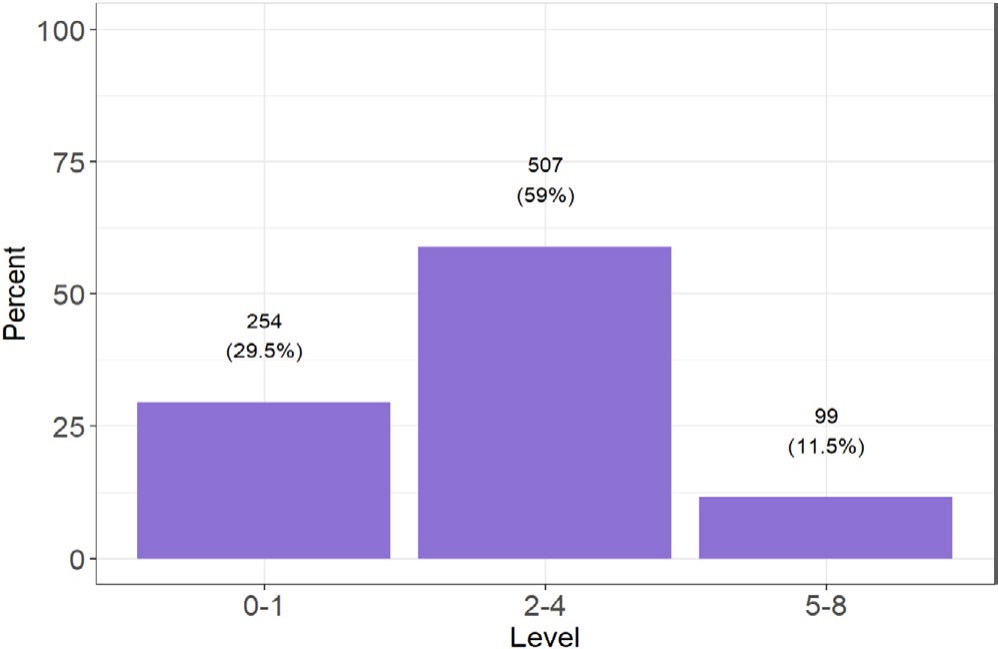
Distribution of study population into main three groups according to CHA2DS2 VASc score.

A total of 919 patients were enrolled in the registry. Of these, 59 patients were excluded due to loss to follow‐up, resulting in a final cohort of 860 patients, all of whom underwent their first pulmonary vein isolation (PVI) procedure. The median age was 66 years, and the median CHA2DS2‐VASc score was 3. The distribution of the CHA_2_DS_2_‐VASc scores is illustrated in Figure 1. The study population was divided into three groups based on their CHA_2_DS_2_‐VASc scores: 0–1, 2–4, and > 5. Baseline and procedural characteristics by score category are presented in Table 1.

**TABLE 1:** CHA_2_DS_2_‐VASc among the general population.

Parameters	*N* = 860 (%)
Age (< 65, 65–74, 75+) (%)	
0	368 (42.8)
1	358 (41.6)
2	134 (15.6)
Female (%)	326 (37.9)
CHF (%)	122 (14.2)
HTN (%)	539 (62.7)
Stroke/TIA/thromboembolic event (%)	95 (11.0)
Ischemic heart disease/vascular disease (%)	168 (19.5)
Diabetes (%)	216 (25.1)
CHA_2_DS_2_‐VASc score levels (%)	
0	108 (12.6)
1	146 (17.0)
2	172 (20.0)
3	197 (22.9)
4	138 (16.0)
5	54 (6.3)
6	34 (4.0)
7	8 (0.9)
8	3 (0.3)
CHA_2_DS_2_‐VASc (median [IQR])	3.00 [1.00, 4.00]

Approximately two‐thirds of the cohort were male and presented with paroxysmal AF. Cryoablation was the predominant ablation strategy, used in 762 patients (88.6%), followed by radiofrequency ablation (RFA) in 42 patients (4.9%), and a combination of cryoablation and RFA in 56 patients (6.5%). Approximately two‐thirds of the patients were treated with antiarrhythmic drugs prior to the procedure. As expected, patients in the higher CHA_2_DS_2_‐VASc score groups were older and more likely to have a history of cardiovascular disease and non‐cardiac comorbidities. More detailed information regarding baseline and procedural characteristics can be found in Table 2 and the appendix (Tables A1, A2, A3, A4, A5, B, C, D).

**TABLE 2:** Basic characteristics of study population.

CHA_2_DS_2_‐VASc score					
*n*	Overall	0–1	2–4	5–8	*p*
	860	254	507	99	
Age (median [IQR])	66.00 [58.00, 72.00]	56.00 [48.00, 62.00]	68.00 [64.00, 73.00]	75.00 [70.00, 78.00]	< 0.001
Male (%)	534 (62.1)	212 (83.5)	281 (55.4)	41 (41.4)	< 0.001
AF classification (prompting ablation): (%)					
Long standing persistent	27 (3.1)	9 (3.5)	12 (2.4)	6 (6.1)	0.252
Paroxysmal	557 (65.1)	189 (75.0)	313 (62.0)	55 (55.6)	< 0.001
Persistent	272 (31.8)	54 (21.4)	180 (35.6)	38 (38.4)	< 0.001
AF duration in year (median [IQR])	3.00 [1.00, 5.00]	2.00 [1.00, 5.00]	3.00 [1.00, 5.00]	3.00 [1.00, 6.75]	0.044
AFL (%)	185 (21.8)	54 (21.6)	104 (20.8)	27 (27.6)	0.405
Attempt at AFL termination (% out of Atrial flutter patients)	91 (49.7)	26 (48.1)	53 (51.5)	12 (46.2)	0.98
Prior anticoagulant type: (%)					
Apixaban	455 (60.6)	82 (48.8)	315 (64.4)	58 (61.7)	0.008
Dabigatran	83 (11.1)	21 (12.5)	52 (10.6)	10 (10.6)	0.573
Rivaroxaban	187 (24.9)	55 (32.7)	111 (22.7)	21 (22.3)	0.024
Warfarin	26 (3.5)	10 (6.0)	11 (2.2)	5 (5.3)	0.403
Antiplatelet type: (%)					
Aspirin	34 (59.6)	12 (85.7)	15 (53.6)	7 (46.7)	0.034
Clopidogrel	22 (38.6)	2 (14.3)	12 (42.9)	8 (53.3)	0.032
Ticagrelor	1 (1.8)	0 (0.0)	1 (3.6)	0 (0.0)	0.98
Rate control therapy (%)	577 (67.2)	147 (58.1)	362 (71.5)	68 (68.7)	0.004
Anti arrhythmic drugs (AAD) (%)	559 (65.1)	160 (63.0)	340 (67.1)	59 (60.2)	0.952
Procedure performed: (%)					
RF ablation	42 (4.9)	7 (2.8)	29 (5.7)	6 (6.1)	0.091
Cryo ablation	762 (88.6)	233 (91.7)	441 (87.0)	88 (88.9)	0.181
Both Cryo ablation & RF ablation	56 (6.5)	14 (5.5)	37 (7.3)	5 (5.1)	0.806

Abbreviations: AF, atrial fibrillation; AFL, atrial flutter.

**FIGURE 2:**
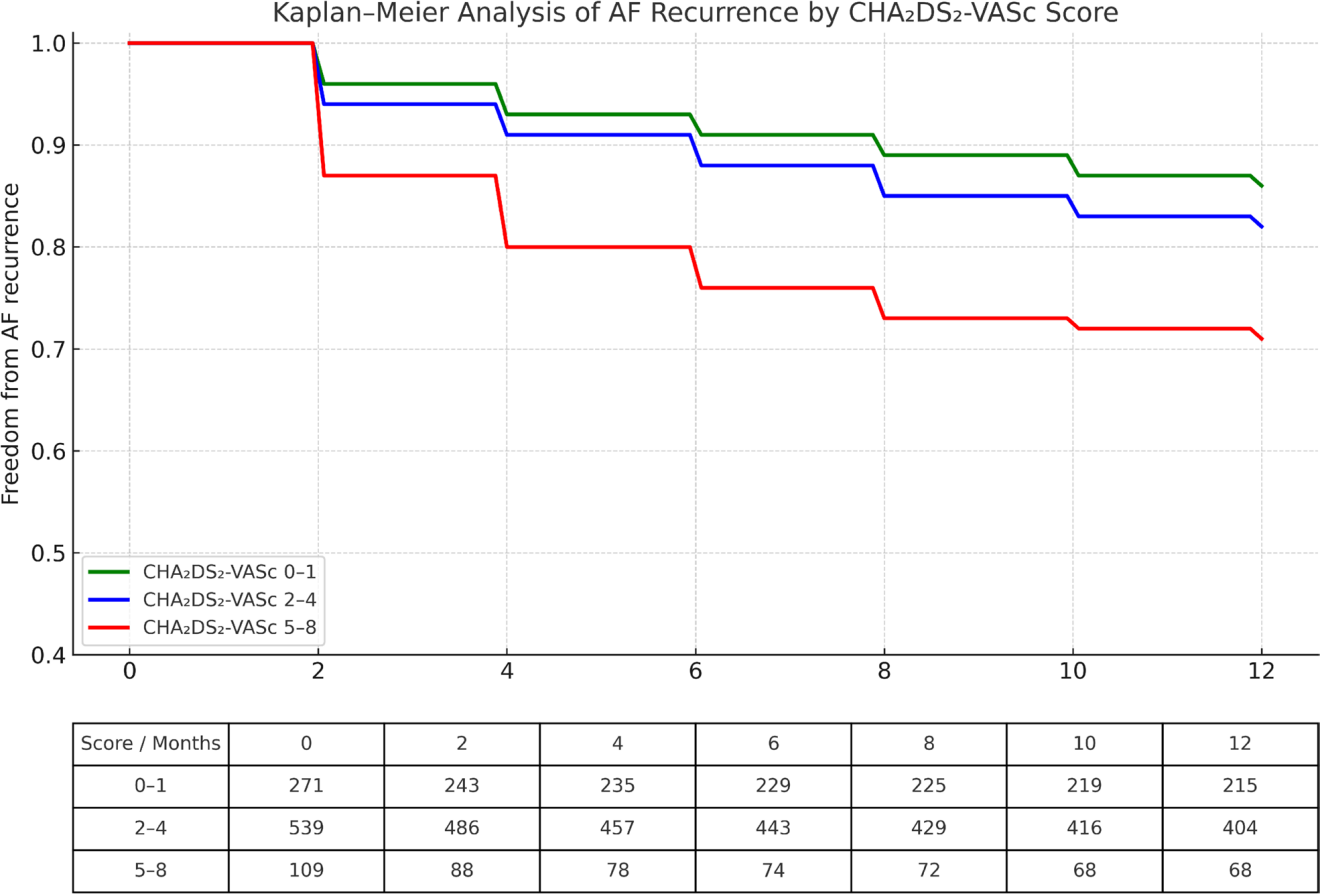
Kaplan–Meier curves demonstrating freedom from atrial fibrillation (AF) recurrence following first‐time pulmonary vein isolation (PVI), stratified by CHA_2_DS_2_‐VASc score groups (0–1, 2–4, and 5–8). Patients with higher CHA_2_DS_2_‐VASc scores exhibited significantly higher recurrence rates over the 12‐month follow‐up period (log‐rank *p* = 0.00024). The table below the graph shows the number of patients at risk at each time point, by score category.

The primary endpoint, defined as AF recurrence within 12 months, occurred in 264 (32%) patients. There was a direct association between the CHA_2_DS_2_‐VASc score and the risk of AF recurrence (Figure 2). In a post hoc analysis, the CHA_2_DS_2_‐VASc score demonstrated modest discriminatory ability for predicting AF recurrence, with an estimated C‐statistic (AUC) of 0.66. One‐year AF recurrence rates were 25.7%, 31.4%, and 51% in the low, intermediate, and high CHA_2_DS_2_‐VASc score groups, respectively (*p* < 0.001) (Table 3). This association remained significant after multivariable adjustment for relevant clinical parameters not included in the CHA_2_DS_2_‐VASc score. Using the low CHA_2_DS_2_‐VASc score group as a reference, there was a significantly higher risk of AF recurrence in the high CHA_2_DS_2_‐VASc score group (HR = 2.88, 95% CI 1.75–4.74, *p* < 0.001) but not in the intermediate CHA_2_DS_2_‐VASc score group (HR = 1.42, 95% CI 0.95–2.12, *p* = 0.13).

**TABLE 3:** Long‐term clinical outcomes for 12 months.

Parameters	Overall	0–1	2–4	5–8	*p*
	*N* = 860 (%)	*N* = 254 (%)	*N* = 507 (%)	*N* = 99 (%)	
Recurrent AF	264 (32.0)	62 (25.7)	153 (31.4)	49 (51.0)	< 0.001
Re Hospitalization within 12 months from admission	191 (22.8)	42 (17.0)	112 (22.6)	37 (38.5)	< 0.001
Adverse cardiac events (%)^a^	18 (2.1)	3 (1.2)	10 (2.0)	5 (5.1)	0.042

^a^ Adverse cardiac events: cardiac death, re‐hospitalization due to acute coronary syndrome, re‐hospitalization due to stroke/transit ischemic attack.

Secondary endpoint events were infrequent. During the 12‐month follow‐up period, nine patients died, including one cardiac‐related death. Major adverse cardiovascular event rates were 1.2%, 2%, and 5.1% in the low, intermediate, and high CHA_2_DS_2_‐VASc score groups, respectively (*p* = 0.042).

## 4 Discussion

The current study was based on the multi‐centre Israeli Catheter Ablation Registry. We demonstrated a direct association between the CHA_2_DS_2_‐VASc score and the risk of atrial fibrillation (AF) recurrence, as well as other adverse outcomes including MACE and re‐hospitalization. However, no significant difference in all‐cause mortality was observed between the groups. Pulmonary vein isolation (PVI) has become the treatment of choice for rhythm control in many patients with AF. When performed in experienced centers, PVI is the most effective treatment for maintaining sinus rhythm and improving symptoms (Krittayaphong et al. 2003; Stabile et al. 2006; Packer et al. 2013; Tzeis et al. 2024).

Several clinical factors serve as preprocedural predictors for PVI success and subsequent AF recurrence after the blanking period. A study of 148 patients who underwent PVI for symptomatic AF with a minimum 6‐month follow‐up identified hypertension and larger left atrial diameter as independent predictors of AF recurrence (Berruezo et al. 2007). In another study involving 1298 patients undergoing PVI for AF, early atrial tachyarrhythmias (EAT) occurred in 40% of patients, while late atrial tachyarrhythmias (LAT) developed in 22% over a follow‐up period of 41 ± 10 months. The study found that longer AF duration, hypertension, and non‐paroxysmal AF were independent predictors of both EAT and LAT (Themistoclakis et al. 2008). Furthermore, a meta‐analysis of 7217 patients undergoing AF ablation reported a 31.2% AF recurrence rate over a 22‐month follow‐up. Patients with persistent AF had a higher risk of recurrence after the first ablation, though this risk diminished with additional procedures. Key predictors of ablation failure were early recurrence within 30 days, valvular AF, and a left atrium diameter greater than 50 mm (D'Ascenzo et al. 2013).

Two distinct scoring systems have been developed to predict AF recurrence following PVI. The first study introduced the APPLE score, which demonstrated enhanced predictive accuracy for AF recurrence compared to the CHADS_2_ and CHA_2_DS_2_‐VASc scores. The APPLE score effectively categorized patients into low, intermediate, and high‐risk groups for recurrence (Kornej et al. 2015). Similarly, the ATLAS score was developed using data from 1934 patients who underwent their first PVI procedure (Mesquita et al. 2018). Five independent predictors of AF recurrence were identified: Age > 60 years, female sex, non‐paroxysmal AF, current smoking, and indexed left atrial volume. These factors enabled the stratification of patients into low, intermediate, and high‐risk categories for AF relapse. However, neither of these scoring methods is widely implemented in routine clinical practice.

Given that the efficacy and safety of PVI can vary among different populations, there is a clinical need for a risk score that can predict procedural success. The CHA_2_DS_2_‐VASc score is a simple and readily available tool that can be easily calculated at bedside.

The association between the CHA_2_DS_2_‐VASc score and PVI success and AF recurrence has been the subject of several studies. One study assessed the predictive value of the CHADS_2_ and CHA_2_DS_2_‐VASc scores for atrial fibrillation recurrence after ablation in 126 patients with paroxysmal AF. Both scores were independently linked to AF recurrence, with a score of ≥ 2 showing the best predictive value, though their sensitivity and specificity were moderate (Letsas et al. 2014). However, the study's findings were based on a relatively small sample size. Another study evaluated the correlation between CHADS_2_, R_2_CHADS_2_, and CHA_2_DS_2_‐VASc scores and AF recurrence after PVI in 2069 patients. While these scores were associated with early and late AF recurrences, their predictive value was relatively low (Packer et al. 2013). The findings suggest that additional factors should be considered in developing better predictive models for AF recurrence. An epidemiological study of 2179 patients undergoing PVI for AF found that both CHADS_2_ and CHA_2_DS_2_‐VASc scores effectively predicted long‐term outcomes, with higher scores indicating increased risk of AF recurrence and major adverse cardiovascular events. The CHA_2_DS_2_‐VASc score demonstrated superior predictive ability compared to the CHADS_2_ score for AF recurrence and related complications (Jacobs et al. 2015). However, the study's reliance on medical records limits the accuracy of outcomes, and its design cannot establish causality or account for variations in procedural approaches.

Our findings are consistent with a recent multicenter study by Rordorf et al. which demonstrated the predictive value of the CHA_2_DS_2_‐VASc score for AF recurrence following cryoballoon ablation in over 3300 patients. In that study, a score ≥ 2 was independently associated with increased recurrence over a 3‐year follow‐up period (AUROC = 0.58) (Rordorf et al. 2023). While both studies affirm the association between this score and post‐ablation outcomes, our analysis adds value by including diverse ablation approaches across a national registry and by incorporating additional outcomes such as MACE and re‐hospitalization.

Furthermore, our study emphasizes the real‐world utility of a score that is already integrated into standard AF management workflows. Unlike more complex imaging‐based predictors (e.g., LAVI or fibrosis imaging), the CHA_2_DS_2_‐VASc score can be used immediately at the bedside to stratify recurrence risk, which may help guide rhythm control strategies and follow‐up planning, particularly in resource‐limited settings.

When evaluating its predictive accuracy, the CHA_2_DS_2_‐VASc score yielded an estimated C‐statistic of 0.66, indicating modest discrimination—consistent with prior studies assessing post‐ablation recurrence. Together, these findings support the use of the CHA_2_DS_2_‐VASc score not only as a thromboembolic risk tool but also as a practical adjunct for outcome prediction in the growing population of patients undergoing PVI.

## 5 Limitations

This study has several limitations that merit consideration. First, it did not include continuous rhythm monitoring, so asymptomatic episodes of atrial fibrillation may not have been detected. Second, the follow‐up period was limited to 1 year. Third, there was no unified post‐procedural management for patients. Additionally, the absence of a standardized post‐procedural management protocol across the participating centers could have introduced variability in patient outcomes due to differences in follow‐up care and antiarrhythmic drug use.

## 6 Conclusion

In conclusion, a higher CHA_2_DS_2_‐VASc score is associated with an increased risk of AF recurrence and adverse cardiovascular outcomes in patients undergoing first‐time PVI. Given its simplicity and routine clinical use, this score may serve as a practical tool to guide risk stratification and personalize post‐ablation follow‐up strategies.

## Author Contributions

Mustafa Gabarin: Conceptualization, data collection, primary manuscript drafting, and final editing. Mahmoud Suleiman: Supervision, critical review, and interpretation of clinical data. Adi Elias: Study design and critical revision of the manuscript. Ibrahim Marai: Patient data acquisition and case validation. Roy Beinart: Contribution to electrophysiological interpretation and manuscript review. Eyal Nof: Clinical input and review of electrophysiological data. Yoav Michowitz: Case contribution and literature review. Michael Glikson: Supervision and expert opinion on device management. Yuval Konstantino: Data interpretation and manuscript feedback. Moti Haim: Contribution to patient management data and manuscript comments. David Luria: Institutional case support and editorial feedback. David Pereg: Manuscript editing and revision support. Avishag Laish‐Farkash: Scientific guidance and critical manuscript review. Alexander Omelchenko: Manuscript editing and figure preparation.

## Conflicts of Interest

The authors declare no conflicts of interest.

## Data Availability

Research data are not shared.

## Appendix A Baseline and Procedural Characteristics for Entire Cohort Study

**TABLE A1:** Baseline demographics and risk factors.

Characteristic	Overall (*n* = 919)	Group 0–1 (*n* = 271)	Group 2–4 (*n* = 539)	Group 5–8 (*n* = 109)
Age (median [IQR])	66.00 [58.00, 72.00]	56.00 [48.00, 62.00]	68.00 [64.00, 73.00]	74.00 [70.00, 78.00]
Male (%)	577 (62.8)	227 (83.8)	304 (56.4)	46 (42.2)
Origin: Israeli Arab (%)	114 (12.7)	51 (19.2)	56 (10.6)	7 (6.5)
Origin: Israeli Jew (%)	774 (86.1)	209 (78.9)	467 (88.6)	98 (91.6)
Origin: Other (%)	3 (0.3)	1 (0.4)	2 (0.4)	0 (0.0)
Origin: Other Israeli (%)	8 (0.9)	4 (1.5)	2 (0.4)	2 (1.9)
BMI (median [IQR])	28.55 [25.40, 32.10]	27.74 [24.81, 30.34]	29.22 [25.79, 32.84]	28.91 [25.95, 32.45]

**TABLE A2:** Medical history and risk factors.

Characteristic	Overall (*n* = 919)	Group 0–1 (*n* = 271)	Group 2–4 (*n* = 539)	Group 5–8 (*n* = 109)
Ischemic heart disease (%)	161 (17.5)	2 (0.7)	107 (19.9)	52 (47.7)
Prior PCI (%)	132 (14.4)	2 (0.7)	83 (15.4)	47 (43.1)
Prior MI (%)	89 (9.7)	2 (0.7)	51 (9.5)	36 (33.0)
Prior CABG (%)	33 (3.6)	0 (0.0)	22 (4.1)	11 (10.1)
Congestive heart failure (%)	135 (14.7)	11 (4.1)	81 (15.0)	43 (39.4)
Hypertension (%)	579 (63.0)	54 (19.9)	422 (78.3)	103 (94.5)
Dyslipidemia (%)	468 (50.9)	66 (24.4)	315 (58.4)	87 (79.8)
Diabetes mellitus (%)	240 (26.1)	10 (3.7)	160 (29.7)	70 (64.2)
Stroke/TIA (%)	85 (9.2)	0 (0.0)	39 (7.2)	46 (42.2)
Chronic lung disease (%)	77 (8.4)	12 (4.4)	51 (9.5)	14 (12.8)
Sleep apnea (%)	174 (19.1)	29 (10.8)	115 (21.5)	30 (27.8)

**TABLE A3:** Rhythm and cardiac history.

Characteristic	Overall (*n* = 919)	Group 0–1 (*n* = 271)	Group 2–4 (*n* = 539)	Group 5–8 (*n* = 109)
AFib symptoms (%)	876 (95.5)	259 (95.6)	509 (94.8)	108 (99.1)
AF classification—Paroxysmal (%)	602 (65.8)	204 (75.8)	336 (62.6)	62 (56.9)
AF classification—Persistent (%)	286 (31.3)	56 (20.8)	189 (35.2)	41 (37.6)
AF duration (years, median [IQR])	3.00 [1.00, 5.00]	2.00 [1.00, 5.00]	3.00 [1.00, 5.00]	3.00 [1.00, 6.25]
Atrial flutter (%)	193 (21.3)	56 (21.0)	109 (20.5)	28 (25.9)

**TABLE A4:** Diagnostic and procedural characteristics.

Characteristic	Overall (*n* = 919)	Group 0–1 (*n* = 271)	Group 2–4 (*n* = 539)	Group 5–8 (*n* = 109)
TTE available (%)	789 (86.8)	224 (83.9)	464 (86.9)	101 (93.5)
LVEF (median [IQR])	60.00 [50.50, 60.00]	60.00 [55.00, 60.00]	60.00 [50.00, 60.00]	57.50 [45.00, 60.00]
LV dysfunction: Moderate (%)	54 (6.9)	8 (3.7)	31 (6.7)	15 (14.9)
LA size (mm, median [IQR])	42.00 [38.00, 47.00]	40.00 [36.00, 44.00]	43.00 [39.00, 48.00]	43.00 [40.00, 47.00]
Mitral regurgitation: Mild (%)	349 (40.1)	70 (27.8)	236 (45.8)	43 (41.3)
Mitral regurgitation: Moderate (%)	83 (9.5)	12 (4.8)	53 (10.3)	18 (17.3)
LA volume (cc, median [IQR])	58.00 [31.25, 82.00]	48.00 [27.00, 60.00]	61.50 [35.00, 84.75]	70.50 [32.00, 95.00]
SPAP (median [IQR])	31.00 [25.00, 38.00]	28.50 [22.25, 31.00]	32.00 [26.00, 39.00]	36.00 [30.00, 47.50]

**TABLE A5:** Medication and interventions.

Characteristic	Overall (*n* = 919)	Group 0–1 (*n* = 271)	Group 2–4 (*n* = 539)	Group 5–8 (*n* = 109)
Anticoagulants used (%)	803 (87.6)	181 (66.8)	519 (96.6)	103 (94.5)
Beta‐blockers (%)	605 (66.0)	159 (58.9)	376 (69.9)	70 (64.2)
Amiodarone (%)	271 (29.5)	53 (19.6)	178 (33.0)	40 (37.0)
Sotalol (%)	32 (3.5)	6 (2.2)	20 (3.7)	6 (5.6)
Digoxin (%)	18 (2.0)	3 (1.1)	12 (2.2)	3 (2.8)
Diltiazem (%)	3 (0.3)	0 (0.0)	2 (0.4)	1 (0.9)
Verapamil (%)	10 (1.1)	1 (0.4)	9 (1.7)	0 (0.0)

**TABLE B:** Cryoablation Procedure Characteristics for the entire study population.

Characteristic	LSPV	LIPV	RIPV	RSPV	RMPV
*n*	871	871	871	871	871
Occlusion grade (Benjamin et al. 2019; Hindricks et al. 2021; Wang et al. 2003; Krittayaphong et al. 2003) (%)					
Occlusion	185 (25.4)	203 (27.9)	207 (28.5)	189 (26.3)	10 (43.5)
Partial occlusion	17 (2.3)	30 (4.1)	44 (6.1)	21 (2.9)	1 (4.3)
Total occlusion	526 (72.3)	495 (68.0)	476 (65.5)	509 (70.8)	12 (52.2)
Pulmonary vein isolation (PVI) status (%)					
No isolation	6 (0.8)	5 (0.7)	10 (1.4)	7 (1.0)	1 (4.0)
Unable to isolate	90 (12.2)	110 (15.1)	149 (20.8)	128 (17.7)	3 (12.0)
Isolated	642 (87.0)	614 (84.2)	559 (77.9)	587 (81.3)	21 (84.0)
Isolation time and temperature					
Time to isolation (sec, median [IQR])	43.00 [30.00, 60.50]	36.00 [25.00, 59.75]	44.00 [30.00, 62.00]	30.00 [21.25, 47.75]	44.00 [37.00, 75.00]
Isolation temperature (−°C, median [IQR])	−38.00 [−42.00, −32.00]	−34.00 [−39.00, −28.00]	−36.00 [−41.00, −31.00]	−35.00 [−40.00, −26.00]	−40.00 [−42.00, −37.50]
Time to −30°C (sec, median [IQR])	29.00 [26.00, 32.00]	30.00 [26.00, 34.00]	30.00 [27.00, 35.00]	28.00 [24.00, 31.00]	29.00 [27.00, 33.00]
Temperature at 60 s (°C, median [IQR])	−41.00 [−45.00, −39.00]	−40.00 [−43.00, −36.00]	−40.00 [−43.00, −36.00]	−42.00 [−46.00, −39.00]	−40.00 [−42.00, −36.00]
−40°C at 60 s (%)	547 (74.7)	428 (59.2)	409 (56.7)	528 (74.7)	12 (75.0)
Minimum temperature (−°C, median [IQR])	−49.00 [−53.00, −45.00]	−47.00 [−51.00, −43.00]	−48.00 [−53.00, −45.00]	−51.00 [−55.00, −46.00]	−45.00 [−48.00, −42.75]
Time to deflation (sec, median [IQR])	48.00 [37.00, 60.00]	42.00 [30.00, 55.00]	42.00 [28.00, 53.00]	48.00 [35.00, 60.00]	33.50 [30.00, 50.00]
Application data					
Number of applications (median [IQR])	1.00 [1.00, 1.00]	1.00 [1.00, 1.00]	1.00 [1.00, 1.00]	1.00 [1.00, 1.00]	1.00 [1.00, 1.00]
Total freeze time (sec, median [IQR])	180.00 [180.00, 240.00]	240.00 [180.00, 240.00]	240.00 [180.00, 240.00]	180.00 [180.00, 240.00]	180.00 [180.00, 240.00]
Phrenic nerve injury during freeze (%)	5 (0.7)	6 (0.8)	33 (4.4)	48 (6.4)	0 (0.0)
PVI confirmation					
PVI confirmed (%)	693 (92.5)	684 (92.4)	679 (92.1)	682 (92.5)	30 (100.0)
PVI not confirmed (Technical issue vs. Physician choice) (%)	4 (10.0)	2 (5.3)	3 (7.5)	3 (7.9)	0 (0.0)

**TABLE C:** Radiofrequent ablation procedure characteristics for the entire study population.

Characteristic	Overall (*n*=111)	Group 0‐1 (*n*=24)	Group 2‐4 (*n*=74)	Group 5‐8 (*n*=13)
3‐D System used (% of RF Ablations)				
Carto	67 (60.9)	11 (45.8)	47 (64.4)	9 (69.2)
EnSite precision	4 (3.6)	0 (0.0)	3 (4.1)	1 (7.7)
None	39 (35.5)	13 (54.2)	23 (31.5)	3 (23.1)
Contact force (%)	67 (60.9)	11 (45.8)	47 (64.4)	9 (69.2)
Multi‐electrode Catheter (%)	62 (56.4)	10 (41.7)	42 (57.5)	10 (76.9)
Multi‐electrode Catheter: Pentaray versus LassoNav (%)	53 (91.4)	9 (100.0)	35 (87.5)	9 (100.0)
Image integration (%)	31 (28.2)	8 (33.3)	21 (28.8)	2 (15.4)
Ablation index				
LSPV (%)	41 (85.4)	7 (70.0)	31 (91.2)	3 (75.0)
LIPV (%)	42 (87.5)	7 (70.0)	31 (91.2)	4 (100.0)
RSPV (%)	39 (84.8)	7 (70.0)	30 (90.9)	2 (66.7)
RIPV (%)	40 (87.0)	7 (70.0)	30 (90.9)	3 (100.0)
Pulmonary vein isolation				
LSPV (%)	45 (90.0)	10 (90.9)	31 (91.2)	4 (80.0)
LIPV (%)	46 (93.9)	10 (90.9)	31 (93.9)	5 (100.0)
RSPV (%)	44 (93.6)	10 (90.9)	30 (96.8)	4 (80.0)
RIPV (%)	46 (95.8)	10 (90.9)	31 (96.9)	5 (100.0)
Additional Arrhythmia Targeted (%)	64 (58.2)	16 (66.7)	42 (56.8)	6 (50.0)
Types of additional Arrhythmia targeted				
Atrial Tachycardia (%)	0 (0.0)	0 (0.0)	0 (0.0)	0 (0.0)
Atypical atrial flutter (%)	11 (10.0)	1 (4.2)	9 (12.2)	1 (8.3)
Typical atrial flutter (%)	51 (46.4)	15 (62.5)	31 (41.9)	5 (41.7)
Other (%)	2 (1.8)	0 (0.0)	2 (2.7)	0 (0.0)
No additional Arrhythmia targeted (%)	46 (41.8)	8 (33.3)	32 (43.2)	6 (50.0)
Additional Ablation lesion (%)	40 (36.0)	7 (29.2)	26 (35.1)	7 (53.8)
Specific additional Ablation lesions				
Non‐PV source (%)	1 (0.9)	1 (4.2)	0 (0.0)	0 (0.0)
Roof line (%)	11 (9.9)	2 (8.3)	7 (9.5)	2 (15.4)
Posterior wall (%)	13 (11.7)	2 (8.3)	9 (12.2)	2 (15.4)
Complex fractionated electrogram (CAFE) (%)	5 (4.5)	1 (4.2)	3 (4.1)	1 (7.7)
Mitral Isthmus (%)	5 (4.5)	1 (4.2)	3 (4.1)	1 (7.7)
Cavotricuspid Isthmus (CTI) (%)	19 (17.1)	4 (16.7)	11 (14.9)	4 (30.8)
Other (%)	6 (5.4)	1 (4.2)	3 (4.1)	2 (15.4)
Superior Vena Cava (SVC) Ablation (% of Non‐PV Source)	0 (0.0)	0 (0.0)	0 (NaN)	0 (NaN)
Block Confirmation for CT Isthmus Ablation (%)	18 (100.0)	4 (100.0)	10 (100.0)	4 (100.0)
Additional Arrhythmia Targeted (Hindricks et al. 2021) (%)	5 (4.6)	1 (4.2)	4 (5.6)	0 (0.0)
Types of additional Arrhythmia targeted (Hindricks et al. 2021)				
Atrial Tachycardia (%)	1 (0.9)	0 (0.0)	1 (1.4)	0 (0.0)
Atypical atrial flutter (%)	3 (2.8)	0 (0.0)	3 (4.2)	0 (0.0)
Typical atrial flutter (%)	1 (0.9)	1 (4.2)	0 (0.0)	0 (0.0)
Other (%)	0 (0.0)	0 (0.0)	0 (0.0)	0 (0.0)
No additional Arrhythmia targeted (Hindricks et al. 2021) (%)	103 (95.4)	23 (95.8)	68 (94.4)	12 (100.0)
Additional specific ablation lesions (%)				
Non‐PV source (%)	1 (0.9)	0 (0.0)	1 (1.4)	0 (0.0)
Roofline (%)	1 (0.9)	0 (0.0)	1 (1.4)	0 (0.0)
Posterior wall (%)	1 (0.9)	0 (0.0)	1 (1.4)	0 (0.0)
CAFE (%)	1 (0.9)	0 (0.0)	1 (1.4)	0 (0.0)
Mitral Isthmus (%)	1 (0.9)	0 (0.0)	1 (1.4)	0 (0.0)
CT Isthmus (%)	4 (3.7)	1 (4.2)	2 (2.8)	1 (7.7)
Other (%)	1 (0.9)	0 (0.0)	1 (1.4)	0 (0.0)

**TABLE D:** procedural and discharge characteristics among cohort study.

Characteristic	Overall (n=919)	Group 0‐1 (n=271)	Group 2‐4 (n=539)	Group 5‐8 (n=109)
Intra or Post‐procedural Events				
DC Cardioversions (%)	279 (30.5)	69 (25.5)	169 (31.5)	41 (38.0)
Heparin Reversal (%)	366 (40.6)	112 (41.5)	201 (38.3)	53 (50.0)
Sheaths Removal (%)	827 (90.7)	239 (88.5)	489 (91.7)	99 (90.8)
If DC Cardioversion				
Immediately After Reversal (%)	293 (35.8)	87 (36.4)	168 (34.9)	38 (39.2)
Immediately using suture (%)	490 (59.9)	142 (59.4)	291 (60.4)	57 (58.8)
Later (%)	35 (4.3)	10 (4.2)	23 (4.8)	2 (2.1)
Cardiovascular events (%)	6 (0.7)	0 (0.0)	5 (0.9)	1 (0.9)
Pericardial Effusion (%)	2 (0.2)	0 (0.0)	2 (0.4)	0 (0.0)
Tamponade (%)	2 (0.2)	0 (0.0)	2 (0.4)	0 (0.0)
Heart Failure (%)	1 (0.1)	0 (0.0)	0 (0.0)	1 (0.9)
Cardiac Arrest (%)	1 (0.1)	0 (0.0)	1 (0.2)	0 (0.0)
Thromboembolic event (%)	1 (0.1)	0 (0.0)	1 (0.2)	0 (0.0)
Peripheral vascular events (%)	13 (1.4)	3 (1.1)	7 (1.3)	3 (2.8)
Neurologic events (%)	7 (0.8)	1 (0.4)	4 (0.7)	2 (1.9)
Pulmonary events (%)	2 (0.2)	0 (0.0)	1 (0.2)	1 (0.9)
Discharge information				
Atrial rhythm at discharge: Sinus (%)	898 (97.7)	264 (97.4)	527 (97.8)	107 (98.2)
Atrial rhythm at discharge: Atrial Tachycardia (%)	3 (0.3)	1 (0.4)	2 (0.4)	0 (0.0)
Atrial rhythm at discharge: AFib (%)	29 (3.2)	7 (2.6)	17 (3.2)	5 (4.6)
Atrial rhythm at discharge: Atrial Flutter (%)	5 (0.5)	1 (0.4)	1 (0.2)	3 (2.8)
Atrial rhythm at discharge: atrial paced (%)	3 (0.3)	1 (0.4)	2 (0.4)	0 (0.0)
Status at discharge: Alive (%)	919 (100.0)	271 (100.0)	539 (100.0)	109 (100.0)
Discharge medications				
Anticoagulants (%)	895 (97.4)	258 (95.2)	532 (98.7)	105 (96.3)
Anticoagulant type (%)				
Apixaban	525 (58.7)	116 (45.0)	347 (65.2)	62 (59.6)
Dabigatran	101 (11.3)	31 (12.0)	56 (10.5)	14 (13.5)
Rivaroxaban	246 (27.5)	103 (39.9)	119 (22.4)	24 (23.1)
Warfarin	22 (2.5)	8 (3.1)	10 (1.9)	4 (3.8)
Antiplatelets (%)	45 (4.9)	6 (2.2)	24 (4.5)	15 (13.8)
Antiplatelet type (%)				
Aspirin	24 (54.5)	4 (66.7)	12 (52.2)	8 (53.3)
Clopidogrel	18 (40.9)	1 (16.7)	10 (43.5)	7 (46.7)
Ticagrelor	2 (4.5)	1 (16.7)	1 (4.3)	0 (0.0)
Rate control therapy (%)	559 (61.0)	147 (54.2)	349 (65.1)	63 (57.8)
Rate control therapy type (%)				
Beta blockers	546 (98.6)	146 (99.3)	339 (98.5)	61 (96.8)
Digoxin	4 (0.7)	0 (0.0)	3 (0.9)	1 (1.6)
Diltiazem	1 (0.2)	0 (0.0)	0 (0.0)	1 (1.6)
Verapamil	3 (0.5)	1 (0.7)	2 (0.6)	0 (0.0)
Anti‐arrhythmics (%)	645 (70.3)	184 (67.9)	391 (72.8)	70 (64.2)
Anti‐arrhythmic types				
Amiodarone	306 (33.4)	62 (22.9)	202 (37.6)	42 (38.5)
Sotalol	29 (3.2)	4 (1.5)	19 (3.5)	6 (5.5)
Propafenone	130 (14.2)	54 (19.9)	67 (12.5)	9 (8.3)
Flecainide	122 (13.3)	47 (17.3)	69 (12.8)	6 (5.5)
Dronedarone	50 (5.5)	14 (5.2)	30 (5.6)	6 (5.5)
Disopyramide	3 (0.3)	0 (0.0)	2 (0.4)	1 (0.9)
